# Geographic variation in lack of food group consumption among children in India: An analysis of change across 720 districts, 2016−2021

**DOI:** 10.1371/journal.pgph.0005077

**Published:** 2026-01-02

**Authors:** Dahyun Park, Rockli Kim, Min-Jeong Shin, Sujata Saunik, S.V. Subramanian

**Affiliations:** 1 Multidisciplinary Research Center for Public Health in Complex System, Korea University, Seoul, South Korea; 2 Associate Professor, Division of Health Policy and Management, College of Health Science, Korea University, Seoul, South Korea; 3 Interdisciplinary Program in Precision Public Health, Department of Public Health Sciences, Graduate School of Korea University, Seoul, South Korea; 4 Professor, School of Biosystems and Biomedical Sciences, College of Health Science, Korea University, Seoul, South Korea,; 5 Independent researcher, Mumbai, Maharashtra, India; 6 Professor of Population Health and Geography, Harvard Center for Population and Development Studies, Boston, Massachusetts, United States of America; 7 Department of Social and Behavioral Sciences, Harvard T. H. Chan School of Public Health, Boston, Massachusetts, United States of America; : St John's Medical College, INDIA

## Abstract

Adequate dietary intake in early childhood is essential for growth and development. However, evidence on geographic disparities and recent trends in food group consumption among young children in India remains limited. This study investigated district-level changes in the consumption of specific food groups among children aged 6–23 months in India. Data from two National Family Health Surveys conducted in 2016 and 2021 of India were used, and a harmonized 720-district geometry was applied to ensure comparability across survey rounds. Multilevel modeling accounting for children nested within communities, districts, and states was used to derive precision-weighted estimates for non-consumption of seven food groups. District-level changes in non-consumption prevalence showed substantial geographic heterogeneity across all food groups. Nationally, non-consumption prevalence declined for most food groups, including other solid, semi-solid, or soft food (−2.0 percentage points), fruits and vegetables (−2.3), dairy products (−1.9), meat/non-vegetarian foods (−1.1). In contrast, non-consumption of grains, roots, and tubers increased (+2.5). Substantial geographic heterogeneity was observed. Between 2016 and 2021, 380 districts experienced declines in non-consumption of other solid, semi-solid, or soft food and 379 in fruits and vegetables, whereas non-consumption of grains, roots, and tubers increased in 335 districts. District-level inequalities remained wide across all food groups. Geographically targeted nutrition interventions are needed to reduce within-country inequalities in young children’s diets.

## Introduction

Dietary intake has been recognized as an important factor of human health and development, directly influencing physical, mental, and social well-being [1]. Globally, the Sustainable Development Goals (SDGs) emphasize the importance of eradicating hunger and malnutrition, with SDG 2.1 targeting universal access to safe, nutritious, and sufficient food by 2030 [2]. Achieving this requires not only eliminating caloric insufficiency but also ensuring adequate intake of diverse, nutrient-dense food groups that support optimal health and development especially in early childhood [3]. An adequate diet, rich in essential food groups such as fruits, vegetables, dairy, pulses, and animal-sourced foods, is a critical determinant of nutritional adequacy [4]. However, food insecurity and deprivation persist in many regions, particularly in low- and middle-income countries, where socio-economic and cultural factors limit access to diverse food items [5]. With India accounting for approximately 21% of the global under-five population, yet over one-third of its children experiencing stunting due to malnutrition, examining food group consumption in India at a disaggregated level is essential to address disparities and inform targeted interventions [6].

Over the past two decades, India’s nutritional landscape has undergone significant changes driven by rapid urbanization, economic growth, and evolving dietary preferences [7]. India has implemented several national initiatives to address food insecurity and malnutrition, including the Public Distribution System (PDS), Integrated Child Development Services (ICDS), the National Food Security Act (NFSA), and Anemia Mukt Bharat. In recent years, Mission Poshan 2.0 has further consolidated national nutrition programs, emphasizing improved complementary feeding, fortified foods, and growth monitoring to strengthen early childhood nutrition service delivery [8]. Despite these changes, the prevalence of food group deprivation remains alarmingly high, particularly among socioeconomically disadvantaged populations [9]. For instance, a substantial proportion of children continue to lack access to high-quality protein sources, such as eggs, dairy, and meat, and essential micronutrient-rich foods, such as vitamin A rich fruits and dark leafy greens [10].

Recent analyses using data from the National Family Health Surveys (NFHS) reveal that while food deprivation has decreased over time, significant disparities persist by wealth, education, and geographic location [11]. According to the Comprehensive National Nutrition Survey, 34.9% of children under five years are stunted, 32.7% are underweight, and 17.3% are wasted, while micronutrient deficiencies remain widespread, with anemia affecting 40.5% of children and iron deficiency affecting 31.9% [12,13]. Nationally representative estimates indicate that infant and young child feeding practices remain suboptimal in India, with only 21% of children aged 6–23 months achieving minimum dietary diversity and approximately 9% meeting the minimum acceptable diet [14].

Interstate variation in food group consumption and nutritional outcomes is shaped by multiple structural factors, including agroecological diversity, market access and food affordability, maternal education, household poverty, cultural dietary norms, and differences in the coverage and quality of services delivered through ICDS and health systems [15–17]. While states like Kerala and Punjab have low rates of malnutrition due to strong public health systems and improved access to diverse food sources, Madhya Pradesh, Bihar, and Jharkhand have substantially higher rates of malnutrition, with more than 50% of children underweight [18]. Understanding the dynamics of dietary changes and disparities at the district level is critical for achieving SDG 2.1 and addressing India’s double burden of undernutrition and diet-related non-communicable diseases [19]. However, most existing studies focus on national or state-level trends, overlooking the rich heterogeneity across districts [20]. This knowledge gap impedes efforts to design localized strategies to improve food security and dietary quality [21].

The objective of this study is to examine district-level variance in the consumption of food groups among children, using the most recent two waves of the NFHS from 2016 to 2021. We further assess changes in these proportions over time, with a focus on geographic disparities. By identifying areas and populations where food groups are consistently underserved, we can contribute to tailored nutrition policies and interventions to improve diet quality and equity across India.

## Materials and methods

### Data and study setting

This analysis used data from the fourth (2015–2016) and fifth (2019–2021) rounds of India’s NFHS, following stratified, two-stage sampling design to ensure representativeness across all states and districts of India’s diverse geographic and socio-economic landscape. In the first stage, communities were selected with probability proportional to size from districts within states. In the second stage, households were randomly sampled from each selected community. For simplicity, the terminal years of each survey are used to refer to the corresponding rounds throughout this study. Both surveys are part of the Demographic and Health Surveys (DHS) Program, which collects data on population health, nutrition, and well-being, including dietary consumption [22]. A detailed description of the survey is available in the NFHS report [23]. The reporting of this study adheres to the STROBE guidelines for observational research. The checklist is provided in the supplementary materials for reference.

### District geometry

To examine district-level variation in dietary quality across India and its changes between NFHS-4 and NFHS-5, we applied a combined district geometry that reflects India’s sub-national geography that are frequent updated due to administrative changes. There were 640 districts during NFHS-4 and 707 districts during NFHS-5. To ensure comparability between survey rounds, we utilized an updated district geometry comprising 720 districts, consisting of 707 districts from NFHS-5 and 13 new districts created in the state of Andhra Pradesh (AP) in April 2022 [24]. This inclusion was necessary, as none of the NFHS-5 districts in AP matched the updated district boundaries. To integrate the new district boundaries, Assembly Constituency (AC) boundaries were linked with the 707-district geometry provided by the DHS Spatial Data Repository [25]. The AC linkage information was obtained from the Chief Electoral Officer [24]. Since AC boundaries are nested within district boundaries, dissolving AC polygons allowed the creation of updated district boundaries [24]. This step resulted in the generation of a 720-district shapefile for NFHS-5. To maintain consistency, the external boundary of the updated shapefile was adjusted to align with the Survey of India’s specifications [26]. To link communities to the updated geometry, we did not make any changes to the PSU to district linkage for the 694 unchanged districts in NFHS-5 and 577 unchanged districts in NFHS-4 and used the PSU to district linkage present in the microdata. For the remaining 23 districts for NFHS-5 and 130 districts that experienced changes in NFHS-4, a PSU-to-district spatial join was performed using GPS coordinates of each PSU to assign them to the updated 720-district geometry. All figures were created by the authors using R (R Foundation for Statistical Computing, Vienna, Austria). Three sources were used to develop the 720-district shapefiles: 1) 707-district shapefile from the DHS Spatial Data Repository (https://spatialdata.dhsprogram.com), which permit use for legitimate academic research under the DHS Program’s Dataset Terms of Use (https://dhsprogram.com/data/terms-of-use.cfm); 2) AC shapefile from Datameet (https://github.com/datameet/maps/commit/7a1c21c2e3f059a19aba1023f64ed7e174462ca4), which are shared under the Creative Commons Attribution 4.0 International license; and 3) the external boundary of India from the Survey of India (https://www.surveyofindia.gov.in/pages/state-maps), with copyright and terms of use available at https://www.surveyofindia.gov.in/pages/copyright-policy. All the underlying shapefiles are publicly accessible and licensed for reuse, ensuring compliance with open access publishing requirements.

### Study population

The study population comprised infants and young children aged 6–23 months who were the most recent births within the two years preceding the survey and were alive and residing with their mothers (aged 15–49 years) at the time of data collection. Food-related information was not collected for children younger than 6 months or older than 2 years. Observations with food-related responses marked as “don’t know” or missing (unreported) were excluded from the analysis. Non-response rates for food-related questions were less than 1% across the surveys [27]. The sample sizes based on the inclusion criteria were 71,538 (2016) and 62,167 (2021). After excluding non-responses to food-related questions (267 in 2016 and 158 in 2021), the final analytic samples were 71,271 (2016) and 62,009 (2021).

### Outcomes

We used food consumption questions to define non-consumption of food groups. Mothers were asked the following question regarding their child’s dietary intake: “Did [Child’s NAME] drink/eat [specific food items] anytime during the day (yesterday) or night (last) 24 h prior to the survey?” Responses included all food items consumed, even if mixed with other ingredients. Both NFHS-4 and NFHS-5 included 18 food-related questions under the category of “Food (Including Milk)” (Table 1). Non-consumption of specific food groups is defined as children who did not consume all foods from each food group in the past 24 hours. The seven food groups included in this study are: Any other solid, semi-solid, or soft food; Fruits and vegetables (Any dark green, leafy vegetables; Any pumpkin, carrots, squash or sweet potatoes that are yellow or orange inside; Any ripe mangoes, papayas, cantaloupe, or jackfruit; Any other fruits or vegetables); Grains, roots, and tubers (Any bread, roti, chapati, rice, noodles, biscuits, idli or any other foods made from grains; Any white potatoes, white yams, manioc, cassava, or any other foods made from roots; Any foods made from beans, peas, lentils, or nuts); Meat/non-vegetarian (Any liver, kidney, heart or other organ meats; Any other meat; Any chicken, duck or other birds; Any eggs; Any fresh or dried fish or shellfish); Dairy products (Any other milk such as tinned, powdered, or fresh animal milk; Yogurt; Any cheese or other food made from milk); Formula food (Infant formula; Any commercially fortified baby food such as cerelac or farex); Liquid food excluding milk (Any other liquids; Juice or juice drinks; Clear broth).

**Table 1:** Prevalence of food group not consumed in the last 24 hours among children in 2016 and 2021.

	Mean (95% CI)		
Food group	2021	2016	Percent point change
Other solid, semi-solid, or soft food	76.81 (75.92–77.71)	78.82 (77.81–79.83)	-2.00 (-2.86–-1.13)
Fruits and vegetables	50.43 (49.54–51.31)	52.69 (51.71–53.67)	-2.26 (-3.06–-1.46)
Grains, roots, and tubers	33.34 (32.57–34.11)	30.80 (30.08–31.53)	2.54 (1.66–3.41)
Meat/non-vegetarian	88.09 (87.28–88.91)	89.20 (88.29–90.10)	-1.11 (-1.70–-0.52)
Dairy products	81.66 (80.91–82.41)	83.53 (82.65–84.40)	-1.86 (-2.53–-1.19)
Formula food	77.23 (76.34–78.12)	78.79 (77.68–79.90)	-1.55 (-2.68–-0.43)
Liquid food (excluding milk)	58.37 (57.33–59.41)	60.47 (59.34–61.61)	-2.10 (-2.94–-1.26)

Note: Prevalence values are presented as percentages with corresponding 95% confidence intervals. The percentage point change is 2021 minus 2016. CI, confidence interval.

### Statistical analysis

The NFHS data were structured hierarchically, with children nested within communities *j*, districts *k*, and states *l.* Given this nested structure, a multilevel model was estimated using a Markov Chain Monte Carlo (MCMC) procedure to determine the district-level prevalence (%) of food groups not consumed in 2016 and 2021 across India. The MCMC procedures follow a Bayesian approach in which prior knowledge is used to maximize a likelihood function [28]. In this analysis, we calculated the priors using iterative generalized least squares (IGLS) with a 2nd-order predictive quasi-likelihood (2PQL) approximation [29]. This was applied to the model $logit\left({Pr}_{ijkl}\right)=\;\beta_{\text{0}\;}+\left(u_{\text{0}jkl}+v_{\text{0}kl}+f_{\text{0}l}\right)$ for each outcomes in each survey round where $\beta_{\text{0}\;}$ represents the constant, and $u_{\text{0}jkl}$, $v_{\text{0}kl}$, and $f_{\text{0}l}$ are the residual differentials for communities *j*, districts *k*, and states *l,* respectively. Priors derived from IGLS were subsequently applied to the MCMC model, with a burn-in of 500 cycles and monitoring of 10,000 iterations. This approach ensured an effective sample size (ESS) greater than 250 for each model. The ESS provides an estimate of the independent samples equivalent to the 10,000 dependent samples generated during the MCMC process [28,30]. Residual estimates were derived using the *runmlwin* command in Stata 18 [31], and the precision-weighted estimates for community-level outcomes were calculated using the equation: exp[$\beta_{\text{0}\;}+\left(u_{\text{0}jkl}+v_{\text{0}kl}+f_{\text{0}l}\right)]/[$1 + exp($\beta_{\text{0}\;}+\left(u_{\text{0}jkl}+v_{\text{0}kl}+f_{\text{0}l}\right)]$.

To analyze district-level changes, decile cutoffs were derived from the 2016 district averages for each outcome. These cutoffs were applied to the 2021 district averages to assess temporal shifts in prevalence. The difference between 2016 and 2021 averages was computed for each outcome, and changes were categorized into seven levels: Substantial Decrease (≤-10.00%), Moderate Decrease (-5.00 to -9.99%), Small Decrease (-2.50 to -4.99%), No Change (-2.50 to 2.49%), Small Increase (2.50 to 4.99%), Moderate Increase (5.00 to9.99%), Substantial Increase (≥10.00%).

### Ethics statement

The NFHS obtained data with informed consent, and the survey protocol, including all questionnaire content, received approval from the Institutional Review Boards of the International Institute for Population Studies and ICF. For this study, the Harvard Longwood Campus Institutional Review Board (IRB) permits researchers to self-assess whether their work requires IRB oversight using the IRB Decision Tool. Based on this tool, our analysis did not qualify as human participant research under regulatory definitions, and thus was deemed exempt from full institutional review.

### Results

**Fig 1:**
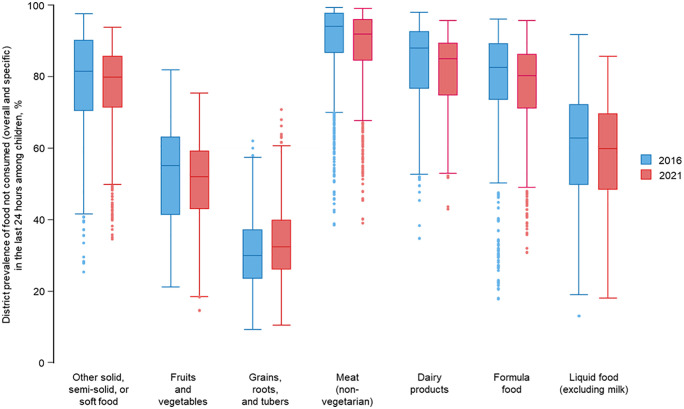
District prevalence of food group not consumed in the last 24 hours among children in 2016 and 2021. Note: Box plots show the distribution of district-level prevalence (%) for each body mass index (BMI) category in 2016 and 2021. The upper and lower bounds of the box represent the 75th and 25th percentiles, respectively. The solid line within the box represents the median (50^th^ percentile).

For children who did not consume any solid, semi-solid, or soft food, the median district prevalence decreased from 81.5% in 2016 to 79.9% in 2021, with the interquartile range (IQR) narrowing significantly from 19.7 to 14.4, suggesting reduced variability across districts (**Fig 1**). The median district prevalence of children who did not consume fruits and vegetables decreased from 55.2% in 2016 to 52.1% in 2021, with the IQR narrowing from 21.8 to 16.3. Conversely, the median district prevalence of children who did not consume grains, roots, and tubers showed an increase from 30.0% in 2016 to 32.4% in 2021, with a slight increase in the IQR from 13.8 to 14.0. In 2016, the median district prevalence of children who did not consume meat among non-vegetarian in India was 94.2%, and 92.0% in 2021, while the IQR increased marginally from 11.1 to 11.5. For dairy products, the median district prevalence of children who did not consume them was 88.0% in 2016, and 85.1% in 2021. The IQR was 16.0 in 2016, and 14.8 in 2021. The median district prevalence of children who did not consume formula food declined slightly from 82.7% in 2016 to 80.3% in 2021, with the IQR narrowing from 15.8 to 15.2. Lastly, the prevalence of children who did not consume liquid food excluding milk decreased from 62.8% in 2016 to 59.9% in 2021, with the IQR narrowing from 22.5 to 21.3.

**Fig 2:**
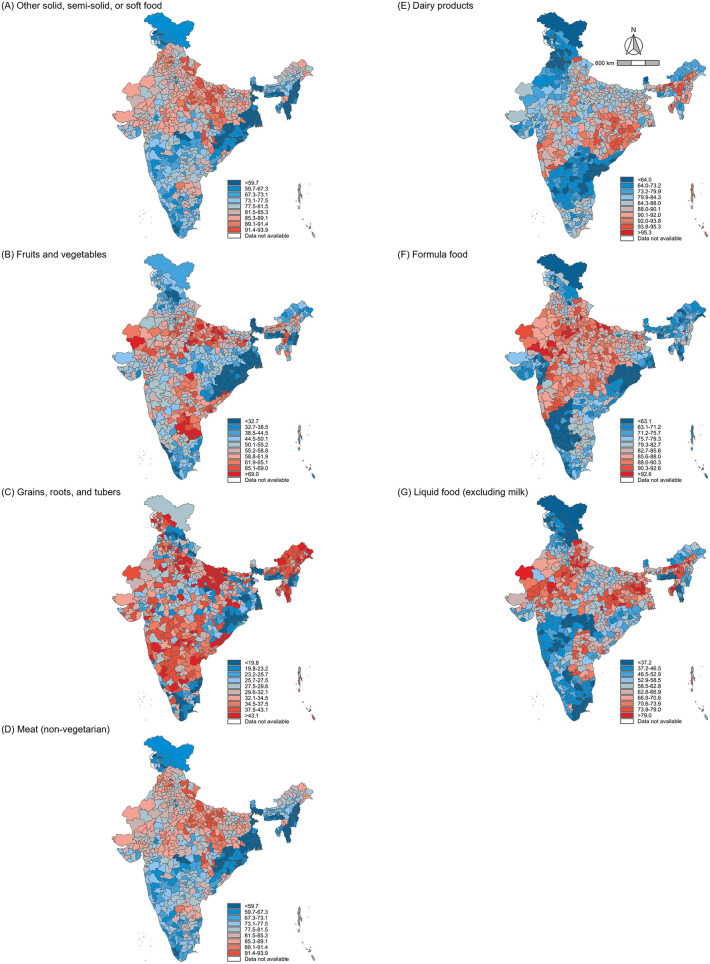
Percent prevalence of food group not consumed in the last 24 hours among children across 720 districts in India, 2021. Note: District-level prevalence estimates for each outcome are shown using decile-based color scales. Decile cutoffs were determined based on the 2016 distribution of each outcome to allow for consistent comparison across time points. The 720-district shapefile was created by the authors as described in the Methods. Fig was created by the authors using R (R Foundation for Statistical Computing, Vienna, Austria).

Fig 2 shows the distribution of district-level prevalence of food groups not consumed among children aged 6–23 months across India in 2021. There are different patterns of geographic distribution in non-consumption for food groups comparing 2016 (S1 Fig). In 2016, the non-consumption prevalence of grains, roots, and tubers was relatively low across most districts in both years but showed an increase in prevalence over the period. In contrast, non-consumption prevalence for meat and dairy products exceeded 80% in large portions of northern and eastern India.

The prevalence of non-consumption of specific food groups in the last 24 hours among children aged 6–23 months across India is summarized in **Table 1**. Approximately 78.8% (95% CI: 77.8–79.8) of children did not consume any other solid, semi-solid, or soft food in 2016, decreasing slightly to 76.8% (95% CI: 75.9–77.7) in 2021. The prevalence of children not consuming fruits and vegetables was 52.7% (95% CI: 51.7–53.7) in 2016 but declined to 50.4% (95% CI: 49.5–51.3) in 2021. Conversely, of children not consuming grains, roots, and tubers showed a slight increase in prevalence, from 30.8% (95% CI: 30.1–31.5) in 2016 to 33.3% (95% CI: 32.6–34.1) in 2021.

**Fig 3:**
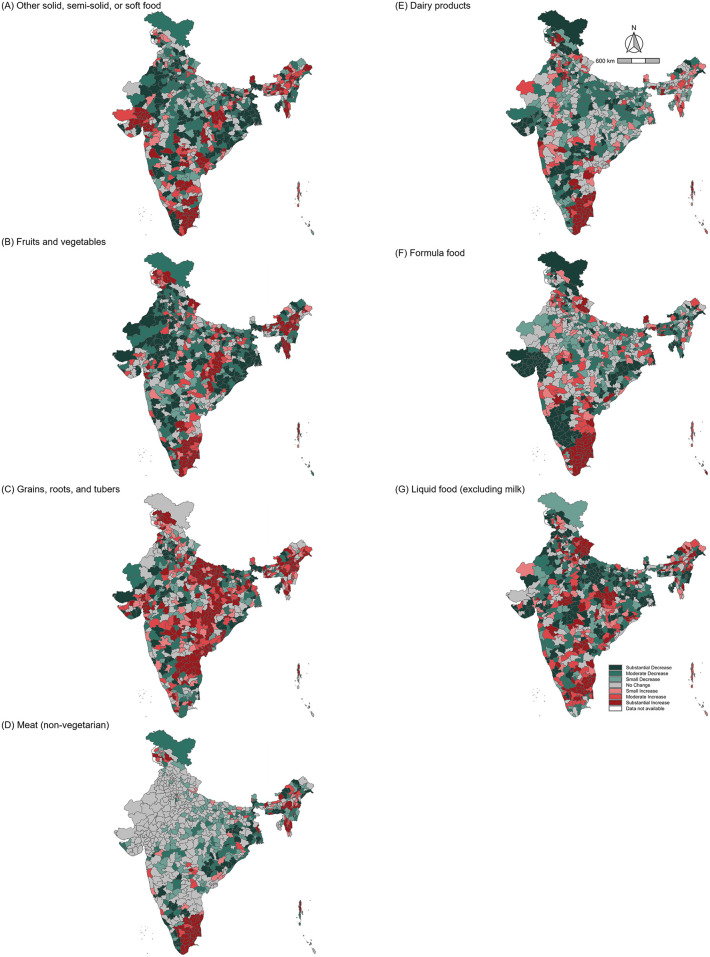
Percentage point change in prevalence of food group not consumed in the last 24 hours among children across 720 districts in India from 2016 to 2021. Note: The percentage point change is 2021 minus 2016. Categories of change are defined as follows: Substantial Decrease (≤-10.00%, dark green), Moderate Decrease (-5.00 to -9.99%, green), Small Decrease (-2.50 to -4.99%, light green), No Change (-2.50 to 2.49%, gray), Small Increase (2.50 to 4.99%, light red), Moderate Increase (5.00 to9.99%, red), Substantial Increase (≥10.00%, dark red). The 720-district shapefile was created by the authors as described in the Methods. Fig was created by the authors using R (R Foundation for Statistical Computing, Vienna, Austria).

**Fig 4:**
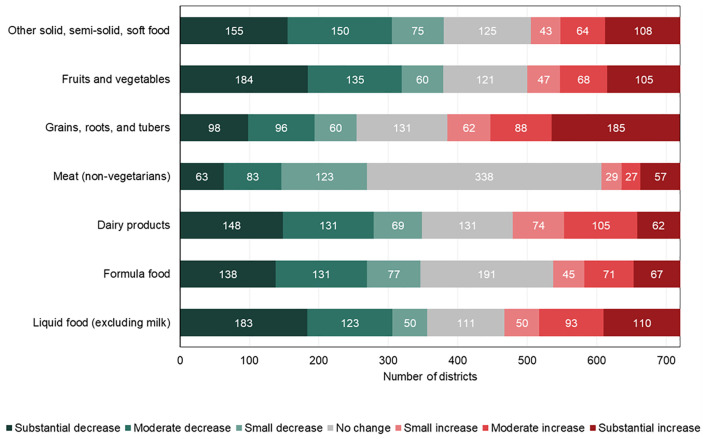
Number of districts with changes in the prevalence of food group not consumed in the last 24 hours among children from 2016 to 2021. Note: The percentage point change is 2021 minus 2016. Categories of change are defined as follows: Substantial Decrease (≤-10.00%, dark green), Moderate Decrease (-9.99 to -5.00%, green), Small Decrease (-4.99 to -2.50%, light green), No Change (-2.49 to 2.49%, gray), Small Increase (2.50 to 4.99%, light red), Moderate Increase (5.00 to 9.99%, red), Substantial Increase (≥10.00%, dark red).

**Fig 3** shows district-level changes in the prevalence of non-consumption for each food group from 2016 to 2021, revealing substantial regional variation. Between 2016 and 2021, non-consumption of grains, roots, and tubers increased in 335 districts and liquid foods in 253, while fruits and vegetables and other soft foods declined markedly (**Fig 4**).

## Discussion

The findings of this study provide important insights into changes in food group consumption patterns among children aged 6–23 months in India, highlighting key trends, disparities, and challenges from 2016 to 2021. First, dietary quality, as measured by the prevalence of non-consumption of specific food groups, showed limited improvement during this period. While there was a slight decrease in the consumption of grains, roots, and tubers, other key food groups, such as fruits, vegetables, and dairy products stagnated or increased. Second, dietary quality varied significantly across districts, with wide disparities in the prevalence of food group consumption. Third, district-level dietary quality improved in several areas but worsened in many others, underscoring persistent inequalities between districts. Districts with the highest levels of food group deprivation in 2016 remained among the worst in 2021.

There are limitations necessary to consider while interpreting the findings. First, these metrics do not assess the frequency or amount of food consumption. However, a lack of diversity in consumption of key food groups during the most critical periods of a child’s development is a useful indicator of a lack of food quality and is a commonly used population-level indicator. Second, while we constructed a harmonized 720-district framework to ensure geographic comparability between NFHS-4 and NFHS-5, a small number of communities could not be linked due to district boundary changes. Thus, while our results may have led to the exclusion or underrepresentation of newly created or conflict-affected regions, potentially limiting generalizability in those areas, previously published studies using the new constituency geometry in Andhra Pradesh show that the standard errors of these constituency estimates are small, indicating that the estimates are accurate [32]. Third, the NFHS has limitations in anthropometric measurements, with inconsistencies in data collection tools and validation affecting estimates. The food consumption questionnaire can contain errors due to recall bias and regional differences in diet, and there is still a need to improve inconsistent training of field interviewers to strengthen data reliability. Fourth, children with missing or “don’t know” responses to food-related items were excluded from the analysis. Although non-response rates were under 1%, listwise exclusion may have introduced minimal selection bias. Similarly, we did not stratify results by social factors such as caste, which are known to influence diet. Lastly, NFHS-5 was conducted during the COVID-19 pandemic, which may have affected food access. However, the dataset does not include detailed survey timing to identify pandemic-related effects. Moreover, since our analysis used only two time points (2016 and 2021), it may not reflect longer-term trends in food consumption. In addition, as a survey-based analysis, this study could not capture the lived experiences, perceptions, or cultural dynamics that shape food choices. These dimensions could be explored in future by investigating using intersectional approaches or mixed-methods research.

Despite these limitations, our findings of this study provide critical insights into the structural, economic, and agricultural factors shaping food group consumption among children aged 6–23 months in India. First, the persistent prevalence of non-consumption of food groups highlights a serious barrier to improved nutrition for children who are most vulnerable during their formative years [33]. This indicator conceptually aligns with the Food and Agriculture Organization’s (FAO) criteria for food insecurity and serves as a valuable measure of extreme food scarcity [34]. Over this period, India underwent major changes, including Goods and Services Tax implementation in 2017, and agrarian reforms during 2020 and 2021, all of which impacted food affordability and access [35,36]. The COVID-19 pandemic exacerbated food insecurity, leading to the expansion of food assistance programs like Pradhan Mantri Garib Kalyan Anna Yojana in 2020, but regional disparities in dietary intake persisted [37]. Given India’s comprehensive policy framework, including the NFSA, which entitles over two-thirds of the population to subsidized food aid [38], the minimal decline in the prevalence of non-consumption across districts remains concerning. Despite India’s economic growth, with GDP increasing by 6.2% in 2024, persistent inflation continues to affect household purchasing power and poor food affordability for low-income populations [39]. The lack of dietary diversity among children reflects insufficient intake of key nutrients essential for healthy growth and development [40]. Diets lacking in animal-source foods, fruits, and dairy are associated with deficiencies in iron, zinc, and vitamin A, which can impair immune function, linear growth, and neurodevelopment [41]. This pattern is especially concerning during early childhood, when nutritional deficits have irreversible long-term effects on health and human capital [42]. These changes underscore the urgent need for targeted interventions to ensure equitable access to diverse and nutritious foods at the district level.

Second, mixed trends in food group consumption highlight critical gaps in achieving dietary quality. While the prevalence of non-consumption of grains, roots, and tubers increased from 30.8% in 2016 to 33.3% in 2021, the other six food groups decreased. These findings reflect significant economic, cultural, and structural barriers to dietary quality. Economic constraints impact dietary quality in India, as the cost of a healthy diet was $3–5 per person per day in 2022, rendering it unaffordable for approximately 74% of the population [43]. Non-consumption of meat, dairy products, and formula food remained still high at 88.1%, 81.7%, and 77.2% in 2021. The low consumption of dairy products and formula food observed in this study may be attributed to the high prevalence of breastfeeding in India, where 84.7% of children are still breastfed at one year of age [44]. However, low maternal dietary quality and inequalities in food distribution within the household still limit the effectiveness of breastfeeding. Interventions that support maternal nutrition, reduce social taboos against breastfeeding, and provide tailored support to young mothers are essential to improving child dietary outcomes. Cultural resistance to animal protein consumption further limits access to eggs, meat, and fish, particularly among wealthier and more educated populations [45]. Religious beliefs strongly influence dietary patterns in India, contributing to low meat, poultry, and fish consumption. About 44% of Hindus and 92% of Jains follow vegetarian diets [46]. Given the widespread vegetarianism in India, there is a need for culturally acceptable strategies that utilize plant-based protein sources, such as pulses and legumes, and dairy products, such as milk and ghee. Indeed, regions with higher dairy consumption, such as Punjab, which has one of the highest per capita milk consumption rates in India (1,271 g/day), have been shown to have better nutritional outcomes [47]. This challenge is compounded by intra-household resource distribution disparities, where women and children often receive less food than other family members [48]. Structural violence contributes to food insecurity because socioeconomic inequalities disproportionately affect marginalized communities’ high rates of poverty, social exclusion, and limited access to resources, limiting dietary quality at the district level. Currently, the SDG primarily tracks overall progress in reducing food insecurity, focusing on caloric sufficiency rather than dietary quality. To address these gaps, food group-specific indicators should be integrated into monitoring frameworks to provide a more comprehensive picture of progress toward improving nutrition and achieving dietary equity [49]. Moreover, agricultural policies in India have historically favored staple crops such as wheat and rice, which may contribute to groundwater depletion and reduced dietary quality [50]. The decline in consumption of millets, once a key component of traditional diets, reflects this shift, despite recent government efforts to promote their nutritional and environmental benefits [51]. Encouraging the integration of millet and other climate-resilient crops into public food programs and consumer markets can improve food security and increase dietary quality, especially in areas of high food burden.

Third, the findings highlight that the geographical disparities vary across food groups. The non-consumption of grains, roots, and tubers reflects their relatively good accessibility as staple foods [45]. Although, even in this case, prevalence increased in several districts, indicating emerging inequities in food access. In contrast, non-consumption prevalence for meat and dairy products is relatively high in northern and eastern India, indicating that cultural and economic barriers to animal sourced food consumption are distributed differently across districts. These findings align with established dietary patterns in India, where northern regions traditionally rely on wheat-based diets with lower inclusion of fruits, vegetables, and animal-sourced foods, driven by both cultural and religious norms [45]. Also, economic constraints and limited market access in many rural areas continue to restrict the availability and affordability of nutrient-rich foods, particularly animal-sourced proteins and dairy products [52]. Therefore, there is a need to strengthen traditional food systems that are locally available, affordable, and culturally accepted, such as dal-rice combinations that complement plant-based proteins, to increase sustainability and economic resilience [53]. While marginal improvements were observed in the overall non-consumption prevalence for many food groups, high-prevalence regions showed little change, indicating persistent geographic inequalities in food access (Fig 3). The district variation in dietary outcomes underscores the need for policies tailored to local contexts [54]. We confirmed the importance of localized factors in shaping geographic inequalities in diet quality, as communities contribute the largest share of variation across all food groups compared to districts and states (S2 Fig). For instance, regions with persistently high non-consumption of fruits, vegetables, and animal-sourced foods require interventions to improve affordability and accessibility. Conversely, districts experiencing worsening trends in staple food consumption, such as grains, roots, and tubers, require targeted support to ensure that basic dietary needs are met. Finally, ongoing conflict-affected regions in India, including the Naxalite insurgency in central and eastern states such as Chhattisgarh, Jharkhand, and Odisha, the separatist movement in the northern region of Jammu and Kashmir in 2019, and ethnic conflicts in northeastern states such as Manipur and Assam, may have face chronic disruption to food systems due to displacement, instability, and limited program delivery [55,56]. These conditions likely contribute to persistent or worsening dietary deprivation in some districts observed in our study.

Fourth, these findings highlight the need for policy approaches that explore the underlying reasons for temporal differences in dietary patterns across regions. Previous studies have pointed to a combination of economic, cultural, and systemic factors that influence changes in food group consumption over time [56]. Rising household incomes and urbanization have driven increased access to certain food groups, such as dairy and fruits, in urban districts, while rural areas continue to face significant barriers due to limited market access and affordability challenges [57]. Changes in food consumption are also influenced by socio-economic factors, particularly education, poverty, lack of access to resources, and market limitations. Previous research has demonstrated that Households with higher education are more strongly associated with improved dietary quality and consumption of essential food groups than household wealth [57]. The findings emphasize the importance of tailoring interventions to local economic realities, including customized affordability and market access, to reduce local food disparities and ensure equitable outcomes. Environmental and systemic factors also play a critical role in these temporal differences. For instance, extreme weather events such as flooding in Madhya Pradesh, Bihar, and Kerala, combined with food supply chain disruptions caused by the COVID-19 pandemic, have disproportionately affected regions already struggling with low dietary quality [45,46]. The uneven impact of government policies, such as the PDS, has also contributed to these disparities [58]. While the PDS has effectively improved access to staple foods like grains, roots, and tubers, its limited inclusion of nutrient-rich foods, such as fruits, vegetables, and protein sources, may explain the slower progress in reducing non-consumption prevalence for these groups. Uneven implementation and quality of service delivery through ICDS, particularly in rural and underserved areas, may contribute to geographic variation in food consumption. Also, Anaemia Mukt Bharat aims to reduce iron deficiency anaemia through supplementation and food fortification, but its effectiveness may be limited in areas with weak infrastructure and poor food access. Similar with the vulnerabilities highlighted in the global context, such as reliance on rain-fed agriculture in sub-Saharan Africa or soil degradation in South Asia, India faces significant barriers to sustainable food production due to groundwater depletion and inefficient agricultural practices. While not examined in this study, climate-resilient agriculture and crop diversification are potential strategies to support food system stability in vulnerable regions [59]. Innovations in agricultural systems, like those promoting lesser-known indigenous crops, can enhance dietary quality while reducing environmental strain. However, progress requires substantial investment, policy coordination, and support for localized, climate-adaptive interventions [60].

The findings of this study underscore the need for a multidimensional policy response that targets the deeper structural drivers of food group deprivation observed across districts. Based on evidence of district disparities, we propose the following approaches that are consistent with the patterns and barriers identified in this study. First, the promotion of regionally adapted traditional diets as affordable, plant-based dietary options that are nutritionally complementary and culturally appropriate is needed. Second, it is essential to expand access to dairy and other culturally accepted protein-rich foods through targeted support and improved supply chain infrastructure, particularly in vegetarian-dominant regions. Third, interventions must prioritize high-burden districts while explicitly addressing inequalities, as some communities continue to experience food deprivation despite aggregate improvements. Fourth, our findings on low dairy and formula intake highlight the importance of supporting maternal nutrition alongside child feeding programs [61]. Finally, the integration of these nutrition strategies within broader social protection frameworks, including poverty, education, and gender empowerment initiatives, will be essential for ensuring sustainable and equitable dietary improvements. These strategies underscore the pressing need to address malnutrition in India and provide a framework for advancing towards Sustainable Development Goal 2.1.

## Conclusion

In conclusion, this study shows that while there was a slight improvement in children’s consumption of certain food groups between 2016 and 2021, persistent district inequalities are limiting progress toward achieving dietary quality in India. Addressing these disparities requires a multi-sectoral approach and geographically targeted interventions that are sensitive to local socio-cultural and economic contexts, focus on equity, and ensure that the most vulnerable populations benefit from nutrition policies and dietary improvements.

## Supporting information

S1 Table Prevalence of food not consumed in the last 24 hours among children in 2016 and 2021. Note: 95% CI, 95% confidence intervals. The percentage point change is 2021 minus 2016.
(XLSX)

S2 Table Variance components from three-level random intercept models of prevalence of food group not consumed in the last 24 hours among children at the state, district, and community levels. Note: Estimates from three-level random intercept logistic regression models. Variance components indicate between-state, between-district, and between-community variation.
(XLSX)

S1 Fig Percent prevalence of food group not consumed in the last 24 hours among children across 720 districts in India, 2016. Note: District-level prevalence estimates for each outcome are shown using decile-based color scales. Decile cutoffs were determined based on the 2016 distribution of each outcome to allow for consistent comparison across time points.
(TIF)

S2 FigGeographic variance partitioned between states, districts, and communities for prevalence of food group not consumed in the last 24 hours among children in 2016 and 2021.Note: Each residual is assumed to be normally distributed with a mean and variance of $u_{\text{0}jkl}$ ~ N(0,$\sigma_{u\text{0}}^{\text{2}}$), $v_{\text{0}kl}$ ~ N(0,$\;\sigma_{v\text{0}}^{\text{2}}$), and $f_{\text{0}l}$ ~ N(0,$\;\sigma_{f\text{0}}^{\text{2}}$), allowing us to calculate the proportion of variation in each outcome attributable to communities, districts, and states by dividing the variance of a given level by the total geographic variation (i.e., for the community level, $\sigma_{u\text{0}}^{\text{2}}$/($\sigma_{u\text{0}}^{\text{2}}$ + $\sigma_{v\text{0}}^{\text{2}}$ + $\sigma_{f\text{0}}^{\text{2}}$) X 100).(TIF)
