# Supplementary figures and images for: Geographic variation in lack of food group consumption among children in India: An analysis of change across 720 districts, 2016−2021
Source: PLOS Glob Public Health. 2026 Jan 2;6(1):e0005077. doi: 10.1371/journal.pgph.0005077 (PMC12758683; doi:10.1371/journal.pgph.0005077)

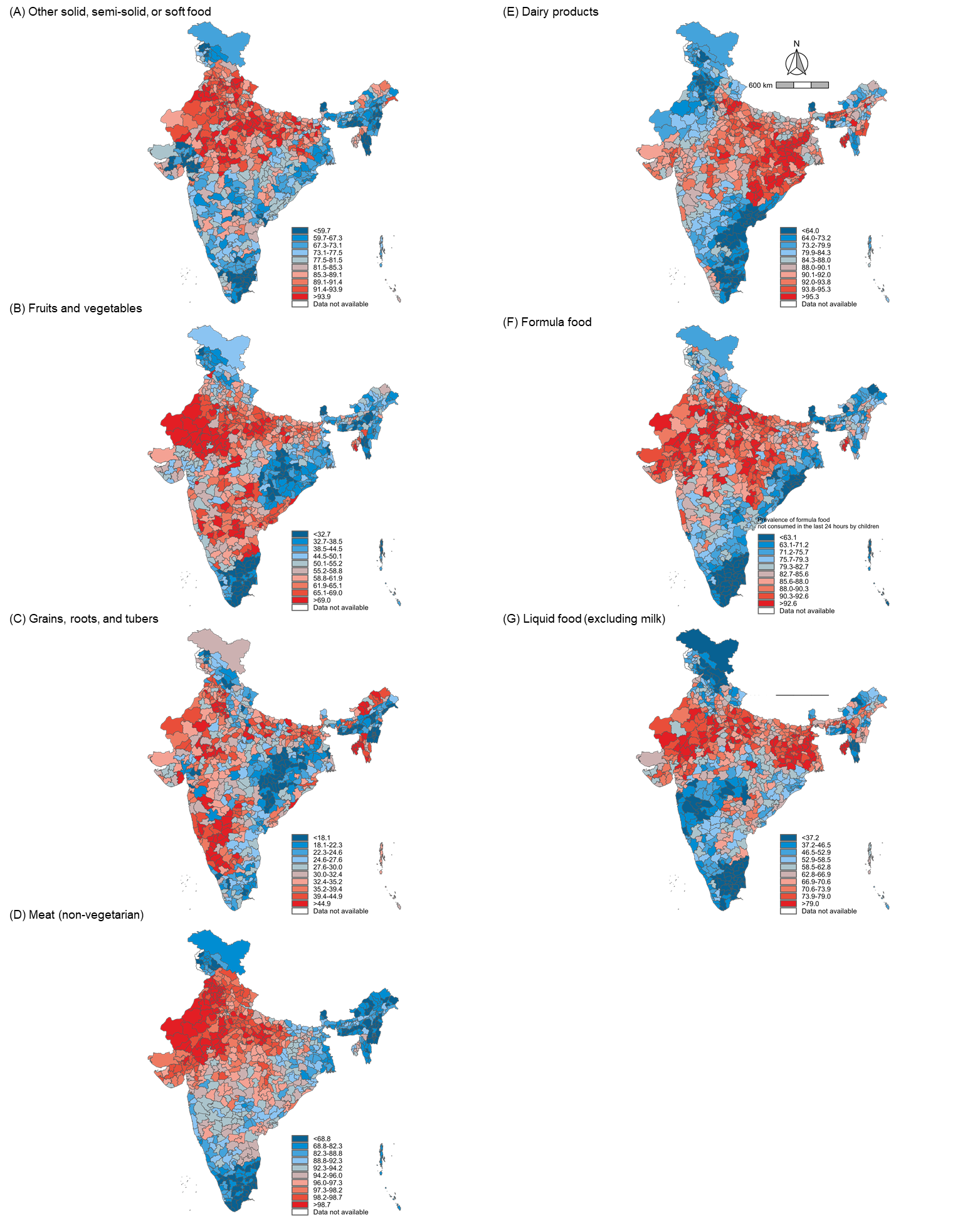

Supplement: S1 Fig — Note: District-level prevalence estimates for each outcome are shown using decile-based color scales. Decile cutoffs were determined based on the 2016 distribution of each outcome to allow for consistent comparison across time points. (TIF) [file pgph.0005077.s003.tif]

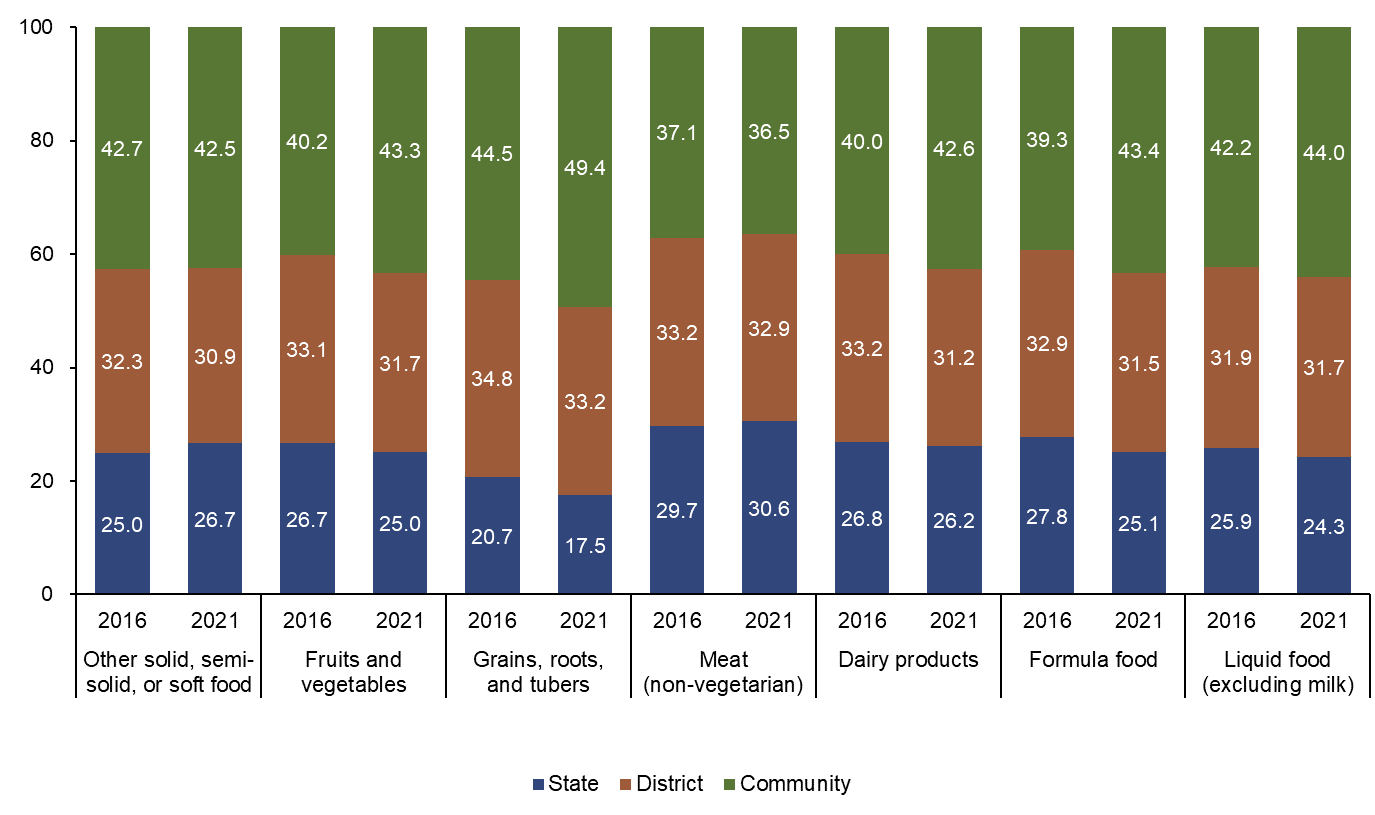

Supplement: S2 Fig — Note: Each residual is assumed to be normally distributed with a mean and variance of u0jkl ~ N(0,σu02), v0kl ~ N(0, σv02), and f0l ~ N(0, σf02), allowing us to calculate the proportion of variation in each outcome attributable to communities, districts, and states by dividing the variance of a given level by the total geographic variation (i.e., for the community level, σu02/(σu02 + σv02 + σf02) X 100). (TIF) [file pgph.0005077.s004.tif]
